# The Medical Practice Act

**Published:** 1881-11

**Authors:** 


### THE MEDICAL PRACTICE ACT.

It is of the first importance that every practitioner of medicine within this State should give attentive heed to the provisions of the lately enacted law to regulate the practice of medicine in Georgia.

In our opinion the law is a good one, and its faithful execution will go far toward relieving the State of much of the disgraceful and fraudulent charlatanry which has hitherto found within her borders so inviting and so lucrative a field for its exercise.

The bill that finally passed both houses of the General Assembly was not the bill indorsed by the Medical Association of Georgia, at its last meeting. That bill, somewhat modified by the house committee who had it in charge, when put upon its passage in the House, was so mutilated by amendments as to be totally deprived of its efficacy; hence, the friends of that bill introduced, as a substitute, the bill which was finally successful, and which possesses the most important features of the original bill; indeed, it is thought by many gentlemen who favored the original bill, to be a decided improvement upon it.

In order to afford a correct understanding of the law, as it now stands, we shall venture to give a synopsis of the act, to which we invite the careful attention of our readers:

1. No person shall practice medicine within this State without a medical diploma, lawfully conferred, by any incorporated college, unless he has been heretofore legally authorized so to do.

2. Every person now practicing must, on or before the first day of next December, register, in a book to be kept by the county clerk, his name, residence and place of birth, and state under what authority he is practicing. If he has a diploma, by whom and when conferred; a license, by whom and when granted; or, if by authority of the State Legislature, when enacted. He must, before registering, exhibit to the clerk an affidavit, duly sworn, setting forth the above facts, that is, his name, residence, place of birth and authority for practicing, whether by diploma, license or legislative enactment, by whom such authority was conferred and the date of the same.

Every person hereafter intending to practice medicine within this State must have a lawful medical diploma, and register, as above set forth, before commencing or offering to practice.

The clerk is entitled to a fee of fifty cents for every registration.

3. Change of residence from one county into another county will necessitate re-registration in the county to which the practitioner removes.

4. The penalty for violating this law, or practicing or offering to practice for fee or reward, without lawful authority, is not less than one hundred dollars or thirty days imprisonment, or both, and not more than five hundred dollars or ninety days imprisonment, or both. One half of fines, when collected, to be paid to the informant.

5. Commissioned medical officers of the United States, dentists in the practice of their profession, and women practicing only midwifery, are exempt from the provisions of the act.

6. All medical boards are abolished, and all conflicting laws are repealed.

It is believed that this act is equitable and free from all unjust discriminations whatsoever. It is destitute of complicated machinery and is scarcely susceptible of misconstruction or abuse, and a fair trial, we doubt not, will establish its unquestionable utility.

The proper enforcement of the law will depend, in a great degree, upon the medical men of the State, and to them we confidently appeal to render the constituted authorities, in the several counties of the State, all necessary assistance to insure its complete and successful operation.

